# Cognitive impairment in candidates for allogeneic hematopoietic stem cell transplantation

**DOI:** 10.1038/s41409-021-01470-z

**Published:** 2021-10-19

**Authors:** Patrick J. Smith, Meagan Lew, Yen Lowder, Kristi Romero, Jillian C. Thompson, Lauren Bohannon, Alyssa Pittman, Alexandra Artica, Sendhilnathan Ramalingam, Taewoong Choi, Cristina Gasparetto, Mitchell Horwitz, Gwynn Long, Richard Lopez, David Rizzieri, Stefanie Sarantopoulos, Keith Sullivan, Nelson Chao, Anthony D. Sung

**Affiliations:** 1 Department of Psychiatry and Behavioral Sciences, Duke University Medical Center, Durham, NC, USA.; 2 Division of Hematologic Malignancies and Cellular Therapy, Department of Medicine, Duke University Medical Center, Durham, NC, USA.

## Abstract

Hematopoietic cell transplant (HCT) is an increasingly common and curative treatment strategy to improve survival among individuals with malignant and nonmalignant diseases, with over one million HCTs having been performed worldwide. Neurocognitive dysfunction is a common and untoward consequence of HCT for many recipients, although few studies have examined the profile of neurocognitive impairments in HCT or their association with clinical features, such as frailty, or the incidence of pre-HCT neurocognitive impairments across all ages, which may influence post-HCT neurocognitive impairments. We examined the pattern and correlates of pre-transplant neurocognitive dysfunction in a prospective sample of adults undergoing HCT. Neurocognition was assessed using the Montreal Cognitive Assessment Battery. Frailty was assessed using the Short Physical Performance Battery. Linear regression analysis was used to examine the associations between neurocognitive performance and frailty. Neurocognitive screening profiles were also examined by partitioning MoCA into domain scores, including Executive Function and Memory. We also examined the associations between neurocognition, frailty, and clinical outcomes, including length of transplant hospitalization and survival. One hundred and ten adults were evaluated across a wide age range (range: 19–75; mean age = 54.7 [SD = 14.1]). Neurocognitive performance tended to fall below published normative levels (mean MoCA = 25.5 [SD = 4.1]), with 17% of participants demonstrating impaired performance compared with medical normative data (MoCA ≤ 22) and 34% exhibiting impaired performance relative to healthy samples (MoCA ≤ 25). Mild impairments (MoCA ≤ 25) were common across age ranges, including middle-aged patients (23% for age < 50; 35% for age 50–60, 41% for age ≥ 60), particularly for items assessing Executive Function. Greater levels of frailty associated with lower neurocognitive screening scores (*r* = −0.29, *P* < 0.01) and Executive Functioning (*r* = −0.24, *P* < 0.01), whereas greater age was associated with poorer Memory performance only (*r* = −0.33, *P* < 0.01). Greater levels of frailty prior to transplant associated with longer length of stay (*β* = 0.10, *P* = 0.046), but were not associated with survival. Neurocognitive impairments are common among adults undergoing HCT and the pattern of performance varies by age. Pre-transplant frailty is associated with neurocognitive functioning and may portend worse post-transplant early clinical outcomes.

## INTRODUCTION

Hematopoietic stem cell transplantation (HCT) is increasingly used as a treatment option for individuals with lymphoma, myelodysplastic conditions, or other forms of refractory cancer [1]. As the population of the United States has aged [2–4], the age of HCT recipients has increased accordingly, with individuals >70 experiencing a threefold increase in the recipient population and routinely evaluated for potential HCT [5]. As the age of recipients has increased, concerns regarding cognitive impairment have become more urgent, with recent consensus documents underscoring the critical nature of assessing cognitive function among HCT candidates [6].

Recent data suggest that increasing age, while critical in the evaluation of HCT candidates, provides an incomplete assessment of potential candidates as chronological and biological age may differentially predict clinical status and outcomes in some HCT recipients [7, 8]. Frailty has emerged as a potentially informative, pre-transplant clinical characteristic linking advanced age to poorer HCT outcomes [9–11]. Greater levels of frailty have been associated with adverse clinical outcomes [1, 12], greater incidence of cognitive impairment, and an increased incidence of postoperative delirium [13]. Emerging evidence suggests that frailty and neurocognitive impairment may have substantial mechanistic overlap, as both frailty and neurocognitive impairment represent manifestations of systemically impaired reserve function [14], with overlapping molecular, immunological, and metabolic pathways of risk [15]. Neurocognitive dysfunction following HCT is an important and understudied clinical outcome, as impairments may worsen quality of life [5] and have been associated with poorer medical adherence, particularly in the domain of executive function [16].

Despite these known associations, few studies have examined both frailty and neurocognition among HCT candidates [17] and none have characterized their overlapping associations. In addition, no studies have delineated impairments in specific cognitive domains (executive function, memory, etc.) among HCT candidates. We therefore examined the associations between frailty, domain-specific neurocognitive performance, and clinical outcomes in a prospectively assessed sample of HCT candidates. We hypothesized that greater frailty would associate with poorer neurocognitive performance on a brief screening measure across domains. We also sought to characterize how these associations might vary across age ranges, hypothesizing that the association between frailty and poorer neurocognition might differ between older and younger HCT candidates due to the high overlap between older age and frailty.

## METHODS

### Study procedures

The protocol was approved as part of a quality improvement initiative for pre-, peri-, and post-HCT optimization within the Duke Adult Bone Marrow Transplant Clinic (Pro00088208). Participants in the present sample were prospectively assessed as part of their pre-HCT evaluation. As part of their routine clinical evaluation, HCT candidates completed assessments of frailty, psychosocial functioning, and underwent a neurocognitive screening assessment. Routine data on patient’s clinical and medical history was also obtained, including medical comorbidities and prior history of cancer treatment.

### Participants

From October, 2017 until March, 2019, 110 participants completed comprehensive assessments that included data on both cognitive function and frailty. Assessments were performed as standard of care among HCT candidates as part of their clinical evaluation. Accordingly, there were no research-defined inclusion and exclusion criteria beyond clinical criteria for seeking and obtaining HCT. Pre-HCT evaluations were conducted by a registered nurse who was trained in the assessment of the SPPB, MoCA, and other clinical assessment metrics. Neurocognitive performance data from the MoCA were also overseen by a study psychologist, who assisted with quality assurance and clinical interpretation. Data pertaining to clinical outcomes were obtained from review of chart records.

### Measures

#### Short physical performance battery (SPPB).

The SPPB was initially developed using data from the Established Populations for the Epidemiologic Studies of the Elderly [18, 19] and has been widely adopted for its clinical utility among transplant candidates [20–24]. The test consists of three components: balance, timed 4 m walk, and chair stands. The standing balance portion required participants to maintain a side-by-side, semi-tandem, and tandem stance for 10 s, with scores ranging from 0 to 4 (maximum score). The chair stands required participants to rise from a chair with arms across their chest for five repetitions. Categorical scores are conventionally used (range 0–4) for the 4 m walk and chair stands are based on normative data from previously reported cohorts [19, 25]. Individuals unable to complete either task received a score of 0. In the present protocol, data were initially collected for identifying the presence of physical frailty using categorical criteria and did not differentiate between balance scores of individuals scoring a 0 (<3 s) or 1 (3–9.99 s) during the tandem balance section. A modified score was therefore used, in which the sum of the three components comprised the final SPPB score with a possible range from 1 to 12, with higher scores indicating lesser levels of frailty. In order to characterize levels of frailty for descriptive purposes, we used SPPB scores of <8 suggestive of frailty, scores of 9–10 suggestive of prefrailty, and scores of 11–12 suggesting that the participant showed no evidence of frailty [26].

#### Montreal cognitive assessment battery.

The MoCA is a brief cognitive screening measure assessing multiple domains of function that has been advocated for use in preclinical or at-risk populations. The MoCA is more sensitive to subtle deficits in executive function in comparison with other more traditional screening instruments, such as the MMSE [27]. The MoCA typically takes <0 min to administer and provides an overall performance score ranging from 0 to 30, as well as domain-specific scores. The MoCA is particularly sensitive to early impairments in memory and executive function, with 83% sensitivity in the detection of mild cognitive impairment (MCI) and 94% in the detection of dementia [28]. In initial publications of the MoCA, a score of 26 was used as the clinical cutoff to identify the presence of MCI, with normative data suggesting that a score <19 was suggestive of dementia [27]. However, following consensus recommendations for the identification of cognitive impairment among adult cancer cohorts [29], more contemporary evaluations of cognitive impairment among individuals with hematological malignancies suggest that a score of ≤25 is optimal to identify individuals performing at ≤ −1.5 standard deviations (SD) below normative levels in at least one domain [30]. Similarly, a score of ≤22 is optimal in the identification of individuals scoring ≤ −1.5 SD below normative levels in two or more domains or ≤ −2.0 SD below normative levels in at least one domain [30]. We therefore used ≤25 and ≤22 to characterize the present cohort.

In the present analyses, we elected to examine domain-specific performance on the MoCA because numerous studies in clinical samples, including transplant patients [16, 31–33], have suggested that executive functioning may be particularly important in its association with selfmanagement behaviors, cognitive decline, and frailty [34–38]. Numerous factor structures have been suggested for the MoCA [39–41], the majority of which delineate between components thought to assess executive function and those assessing memory [41–46]. For secondary analyses of cognitive domains, MoCA scores were reduced into subdomains using principal axis factor analysis available within PROC FACTOR (data not shown). Due to high correlations between tests of executive function, attention, and language, these subtests were combined into one domain and memory recall was used as a separate outcome. We therefore utilized a two-factor grouping within the present study: (1) Memory (Word-List Recall, Orientation, and Naming) and (2) Executive Function/Attention factor (Trial-Making, Cube Copy, Clock Drawing, Digit Span, Auditory Vigilance, Serial Subtraction, Auditory Repetition, Phonemic Fluency, and Verbal Abstraction).

#### Clinical characteristics and outcomes.

Clinical characteristics and outcomes were obtained prospectively. Background clinical factors, including age, gender, disease type, and body mass index (BMI) were obtained from patient clinical assessments at the time of their evaluation for HCT. Treatment regimen was obtained from clinical records and analyzed as either high (myeloablative) or low (all other therapies) intensity treatment. Index HCT hospitalization length of stay (LOS) and the presence of acute graft vs. host disease (aGVHD) were also obtained from clinical records.

### Statistical analyses

General linear modeling was used within SAS 9.4 to examine associations in MoCA score and SPPB, adjusting for age, education, gender, disease type and BMI. A significance level of *P* ≤ 0.05 was used for all analyses. Unadjusted analyses of relationships between background characteristics, frailty, and neurocognitive function were accomplished using simple correlation. Linear regression was used to characterize the associations between SPPB and MoCA scores (PROC REG). Finally, due to the small number of individuals who did not undergo HCT, comparison of participants who did and did not undergo HCT was conducted using *t*-test without additional adjustment. We also examined the associations between SPPB and MoCA scores with transplant LOS and overall survival (OS). Analyses examining LOS utilized generalized estimating equations with a negative binomial distribution, with LOS serving as the outcome adjusting for age, BMI, disease type, conditioning regimen, and aGVHD (PROC GENMOD). In order to account for individuals who died during their index transplant hospitalization, these individuals were given an imputed LOS to reflect a ‘worse’ clinical outcome without biasing model estimates due to a lower LOS. Overall survival (OS) was examined using a proportional hazards modeling approach, adjusting for the same covariates as our LOS model. For both LOS and OS, total MoCA score and SPPB were examined as predictors within parallel models.

## RESULTS

### Background and clinical characteristics

Demographic and clinical characteristics of the sample are shown in Table 1. As shown, participants tended to be middle-aged or older, Caucasian, and male. A substantial minority of participants exhibited cognitive impairment, with 35% exhibiting at least mild impairments based on conventional cut-offs (total MoCA score ≤ 25) in outpatient settings and 17% exhibiting more moderate impairments based on more contemporary, stringent criteria (total MoCA score ≤ 22).

Characterization of frailty and neurocognitive impairments across age groups within the sample revealed that both frailty and neurocognitive impairment were common even among middle-aged participants. The prevalence of neurocognitive impairments was 17% for moderate-to-severe levels (total MoCA score ≤ 22) across the entire cohort and 35% for mild levels of impairment (total MoCA score ≤ 25) across the entire cohort. Examination of the prevalence of mild impairments by age range demonstrated that mild impairments were observed in 23% of participants aged <50, 35% of participants aged 50–60, and 41% of participated aged 60 years or older.

### Background characteristics and cognitive function

Examination of demographic factors revealed greater age exhibited a modest association with worse total MoCA scores (*r* = −0.20, *P* = 0.045). No other demographic factors were associated with cognitive function. Examination of individual MoCA domains (Executive Function and Memory) revealed that these associations differed markedly between cognitive domains. Greater age was strongly associated with Memory recall performance (*r* = −0.33, *P* < 0.001) but was not associated with Executive Function (*r* = −0.07, *P* = 497). In contrast, education was associated with Executive Function performance (*r* = 0.27, *P* = 0.004) but not with Memory recall (*r* = −0.10, *P* = 0.316).

### Frailty and cognitive function

**Fig. 1:**
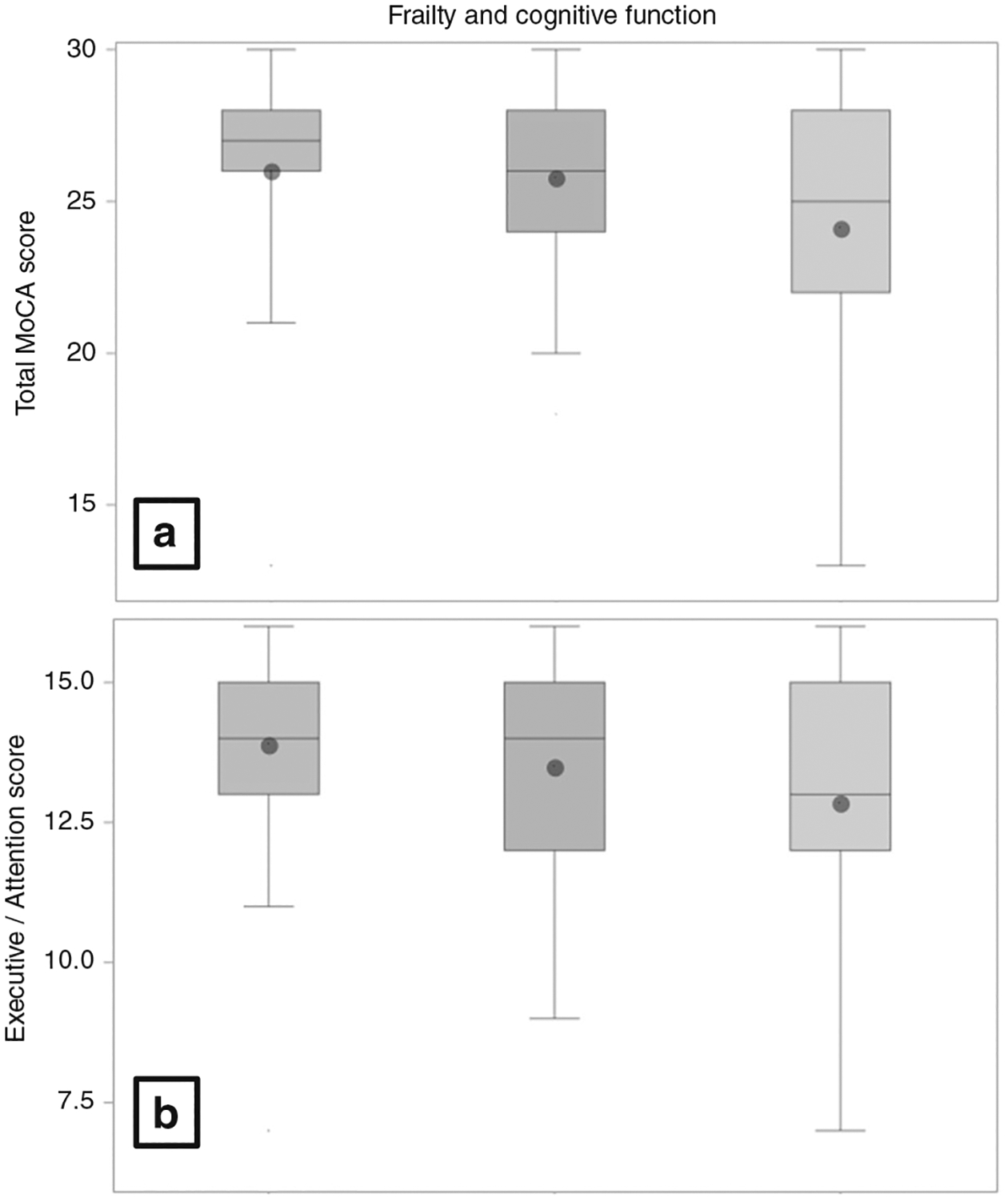
Frailty and cognitive functioning. Figures show levels of cognitive functioning across frailty levels, including participants without frailty (left), pre-frailty (middle), and frailty (right).

Nearly one-third of the sample could be characterized as frail using conventional, community-based cutoffs (≤9; *n* = 32 [31%]). Interestingly, examination of frailty levels across age groups was less consistent than that observed for cognitive impairment, prevalence rates of 27% (≥65), 35% (50–65), and 29% (<50) across age groups. Greater levels of frailty did not associate with any demographic factors, including age (*r* = 0.16, *P* = 0.105). In contrast, greater levels of frailty were strongly associated with poorer cognitive function (total MoCA score: *r* = −0.29, *P* = 0.002) (Fig. 1). Examination of cognitive domains revealed that frailty was most strongly associated with Executive Function (*r* = −0.24, *P* = 0.013) but demonstrated only a weak trend to associate with Memory (*r* = −0.18, *P* = 0.074).

In order to examine the independent associations between frailty and cognitive function, we conducted additional regression analyses controlling for age, education, gender, and disease type (Table 2). Regression analyses revealed that greater frailty continued to be associated with worse cognitive function after accounting for background and clinical characteristics (*β* = −0.28, *P* = 0.003). Follow-up analyses revealed that this association was primarily driven by Executive Function (*β* = −0.28, *P* = 0.003), whereas Memory recall was not associated with frailty levels (*β* = −0.17, *P* = 0.078).

### Frailty, cognitive function, and clinical outcomes

Across the cohort of patients evaluated, 64 (59%) received HCT during the study time period. Twenty-six patients (24%) were ultimately declined or otherwise decided not to pursue HCT. Specifically, six patients did not follow-up with the program following their evaluation, seven died, five patients stabilized medically and/or delayed their transplant, four were declined for psychosocial indications, and four could not be transplanted due to progressive worsening disease status. A remaining 19 patients have completed their evaluation but have not yet been transplanted. Increased levels of frailty (*P* = 0.044) at initial evaluation associated with an increased likelihood of not going on to HCT, with a similar trend for MoCA scores (*P* = 0.092). In contrast, participants who did not undergo HCT did not differ on any other background or clinical characteristic, including age (*P* = 0.986).

Examination of medical outcomes revealed that greater frailty levels were associated with longer LOS. Specifically, greater frailty was associated with longer LOS (*β* = 0.10, *P* = 0.046). In contrast, frailty was not associated with differential OS (HR = 1.45 [0.81, 2.57], *P* = 0.210). In contrast, performance on the MoCA was not associated with LOS (*β* = −0.01, *P* = 0.523), nor was the total MoCA score associated with differential OS (HR = 1.24 [0.87, 1.78], *P* = 0.233).

## DISCUSSION

Results from the present analysis confirm that cognitive impairment and frailty are common among pre-transplant patients and that greater frailty levels associated with poorer cognitive function on a brief screening measure. We found that more than a third of HCT candidates met criteria for mild impairments (total MoCA score ≤ 25) and that nearly one in five continued to meet criteria for more moderate impairment using more stringent cutoffs (total MoCA score ≤ 22). Notably, these associations persisted after accounting for background and demographic characteristics, suggesting that frailty may be an important biobehavioral risk marker independent of its overlay with chronological age or disease type.

Prior studies have suggested that cognitive impairment is common among pre-transplant HCT patients and may associate with adverse clinical outcomes [6]. Indeed, a recent consensus review noted that cognitive impairment is observed in up to 60% of HCT patients prior to transplant depending on the assessment modality, definition of impairment, and timing of assessment with regard to clinical treatment course [47]. The vast majority of studies have been conducted after HCT, however, and studies limiting assessments to pre-transplant reported lower levels of impairment with similarly variable estimates of prevalence [47]. For example, in their meta-analytic review Phillips et al. found that pre-HCT impairments ranged in prevalence from 12 to 89% depending on the methodology of assessment and definition of impairment. Notably, impairments were most common on tests requiring greater psychomotor speed, attention, and executive function, which is consistent with the present findings suggesting that executive dysfunction may be more prevalent than global cognitive impairment. This parallels our own work [31, 48, 49] and that of others [33, 50] in solid organ transplant groups suggesting that impairments in executive function and processing speed are prevalent and may associate most strongly with biomarkers of frailty.

Frailty has also gained increasing attention for its relevance in HCT candidates, with recent data suggesting that frailty is common [10, 11] and associated with clinical outcomes [7, 12]. Similar to observed data for cognitive function, frailty estimates suggest that it is prognostic among non-geriatric candidates, and appears to associate with clinical outcomes above and beyond other demographic or clinical factors. For example, in a sample of 203 middle-aged and older adults undergoing HCT, Muffly et al. [8] found that greater levels of frailty predicted post-transplant mortality and, although this association was strongest in older recipients, the risk associated with frailty was present in younger recipients as well.

Despite the emerging importance of both cognitive impairment and frailty, no studies, to our knowledge, have found a link between these factors among adults undergoing HCT. Greater frailty is well-known to associate with cognitive impairment in geriatric samples, with increasing levels of frailty associating with a greater incidence of developing MCI, Alzheimer’s disease, and dementia [51, 52]. Moreover, targeting frailty for treatment using lifestyle modification has gained interest as a means of reducing the risk of cognitive impairment [53–55]. Although the mechanisms linking frailty, cognitive impairment, and dementia are actively under investigation [51], it has been suggested that some elements of frailty may be modifiable [54] and therefore could be optimized prior to transplant to improve transplant-related outcomes. Indeed, numerous investigative teams are actively examining whether ‘prehabilitation’ approaches among transplant recipients might hold potential to decrease preoperative levels of frailty to improve post-surgical outcomes [56–58]. While these nascent approaches are still being developed for HCT patients [56, 57], numerous randomized trials support the utility of this approach to improve frailty in other medical populations [59–65].

The present study must be viewed with several limitations in mind. First, the present analyses were conducted on a relatively small and select number of HCT recipients. Second, we examined these associations during pre-transplant assessments and did not collect comprehensive, longitudinal data to assess whether improving frailty could improve clinical outcomes, such as reducing the risk of delirium, post-transplant cognitive impairment, or clinical outcomes (i.e., GVHD or survival). Future studies should therefore examine these associations in larger patient cohorts, as well as examining whether improvements in frailty confer improvements in cognitive function and associated outcomes. Third, although there is no current consensus as to the most appropriate screening methodology for cognitive impairment in HCT samples [5], we are limited by our relatively brief assessment battery. Future studies would also benefit from collecting a more comprehensive assessment of cognitive functioning across multiple domains, using cognitive test instruments calibrated to detecting impairments among cancer patients [29, 30]. Finally, our assessment of clinical outcomes was also limited to the perioperative time period, which likely limited our power to detect meaningful associations between cognitive function and clinical endpoints. Future studies incorporating longer follow-up of clinical events will be important to fully characterize the relationships between cognitive function, frailty, and clinical outcomes among HCT recipients.

In conclusion, results demonstrated that cognitive impairment and frailty are common among pre-HCT patients and that greater levels of frailty associate with worse cognitive function, particularly executive function. In addition, these associations persisted after accounting for background and clinical characteristics, suggesting that both cognitive function and frailty may provide important information to inform pre-HCT assessments.

## Figures and Tables

**Table 1.** Demographic and clinical characteristics of the sample.

Variable	
Age, years	54.7 (14.1)
Primary disease type, *n* (%)	
Leukemia	52 (47%)
Myelodysplastic syndrome	22 (20%)
Other	36 (33%)
Gender, Female	40 (36%)
Race, Caucasian	83 (75%)
Body mass index	29.1 (6.5)
Short Physical Performance Battery, Score	10.9 (1.4)
Non-Frail (SPPB ≥ 11)	50 (48%)
Pre-Frail (SPPB 9–10)	35 (34%)
Frail (SPPB < 8)	19 (18%)
Montreal cognitive assessment battery	
Total score	25.5 (4.1)
Moderate-to-Severe Impairment (≤22), *n* (%)	19 (17%)
Mild Impairment (≤25), *n* (%)	38 (35%)
Memory/Orientation score	11.9 (1.5)
Executive Function/Attention score	13.6 (2.2)

Values represent mean (standard deviation) unless otherwise indicated. Individuals with leukemia include acute lymphocytic leukemia, acute myelogenous leukemia, chronic myelogenous leukemia, and chronic lymphocytic leukemia. Individuals classified as having ‘other’ primary diseases included chronic granulomatosis, Langerhan’s cell histiocytosis, X-linked Chronic Granulomatous Disease, and aplastic anemia, among others.

**Table 2.** Results from our multivariate regression model characterizing the association between frailty and cognitive function.

Variable	Standardized estimate	Unstandardized estimate	95% Confidence interval	*P* value
Age	−0.17	−0.05	−0.11, 0.01	0.088
Education	0.15	0.37	−0.10, 0.83	0.115
Male Gender	0.10	0.86	−0.73, 2.44	0.287
Myeloma vs. Leukemia	−0.13	−1.32	−3.37, 0.73	0.203
Other Disease vs. Leukemia	−0.14	−1.24	−3.00, 0.53	0.167
SPPB Score	0.28	0.86	0.29, 1.42	0.003

Total MoCA score served as the outcome, adjusting for age, education, gender, and disease, with SPPB score as the predictor of interest. Disease characteristics were modeled as dummy variables with lymphoma serving as the reference group.
